# Supplementary material for: Assessment of the Utility of Gene Positioning Biomarkers in the Stratification of Prostate Cancers
Source: Front Genet. 2019 Oct 17;10:1029. doi: 10.3389/fgene.2019.01029 (PMC6812139; doi:10.3389/fgene.2019.01029)
Supplement: Supplementary file 8 [file Image_1.pdf]

Supplementary Figure 1

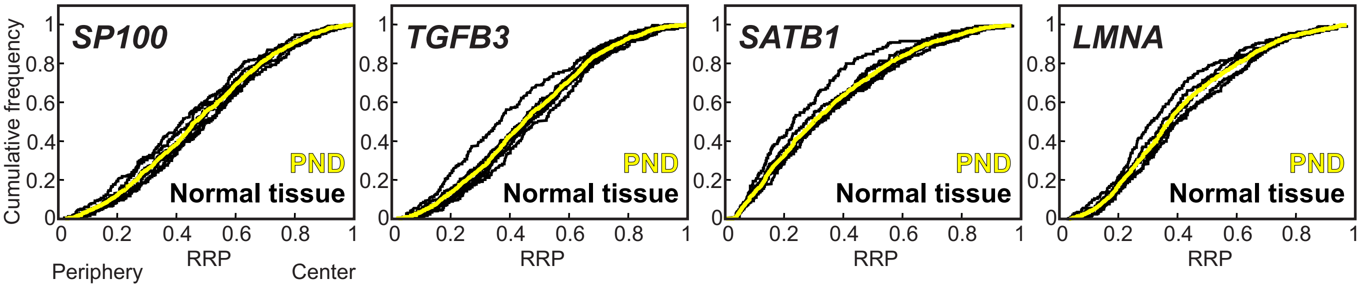

Supplementary Figure 1. Pooled normal distribution. Cumulative RRDs for individual normal prostate tissue (black) and the pooled normal distribution (PND; yellow), generated from combining all normal nuclei for a given gene into a single dataset.
