# Supplementary material for: Assessment of the Utility of Gene Positioning Biomarkers in the Stratification of Prostate Cancers
Source: Front Genet. 2019 Oct 17;10:1029. doi: 10.3389/fgene.2019.01029 (PMC6812139; doi:10.3389/fgene.2019.01029)
Supplement: Supplementary file 9 [file Image_2.pdf]

Supplementary Figure 2

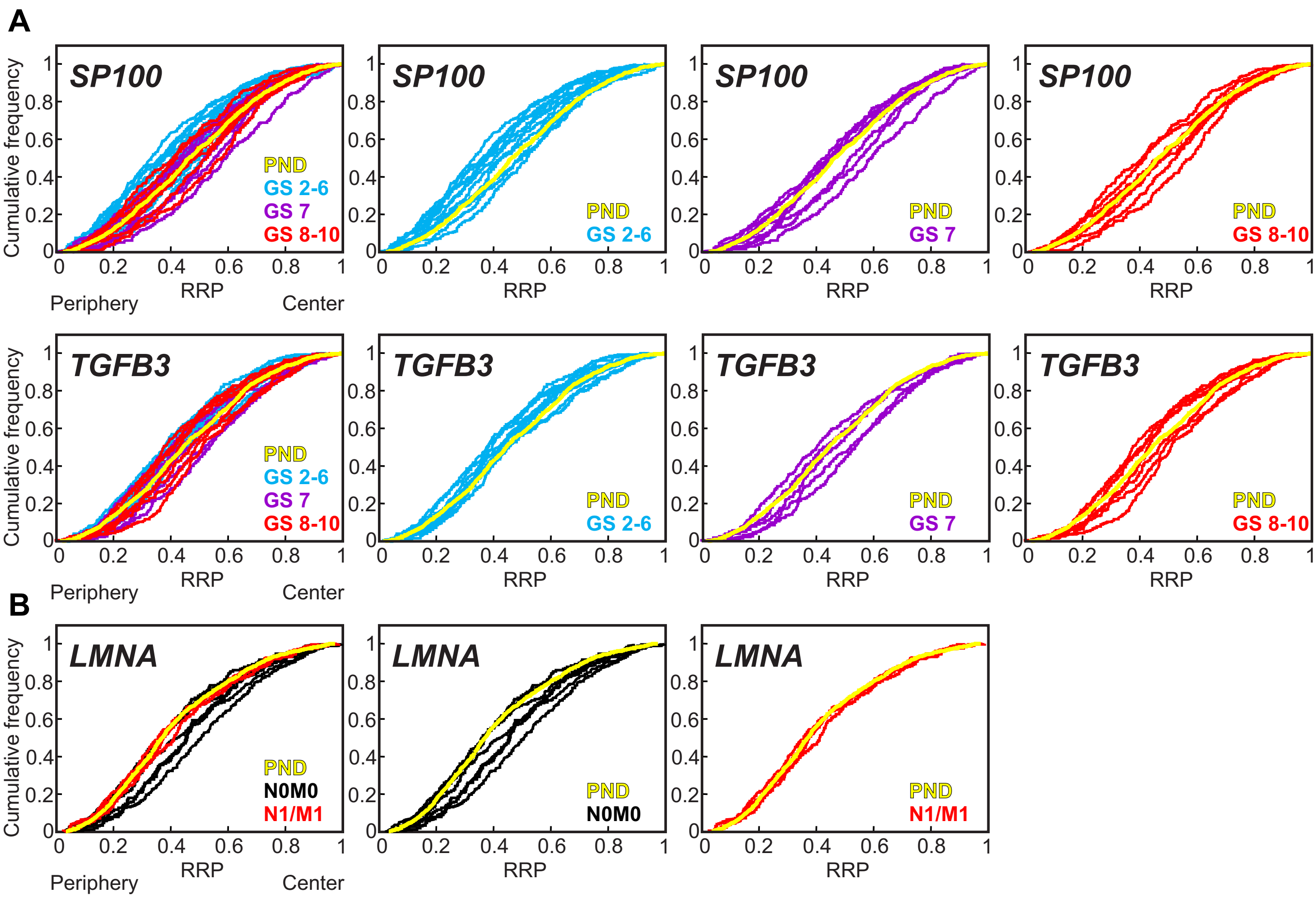

Supplementary Figure 2. Sub-type specific spatial gene repositioning in cancer. (A) Cumulative RRDs for *SP100* and *TGFB3* in prostate cancer tissues are color coded according to Gleason score: Gleason score 2-6 (blue), Gleason score 7 (purple) and Gleason score 8-10 (red). **(B)** Cumulative RRDs for *LMNA* in prostate cancer tissues are color coded according to metastatic status: Non-metastatic (N0M0) cancers (black), metastatic (N1/M1) cancers (red). In all graphs the pooled normal distribution (yellow) is included for comparison. GS, Gleason score; RRP, relative radial position.
